# Experimental study on the control form of fin stabilizer at zero speed

**DOI:** 10.1371/journal.pone.0216395

**Published:** 2019-05-17

**Authors:** Songtao Zhang, Peipei You, Peng Zhao, Lihua Liang, Rubing Li

**Affiliations:** 1 College of Automation, Harbin Engineering University, Harbin, Heilongjiang, China; 2 China Ship Development and Design Center, Shanghai, China; Universita degli Studi della Tuscia, ITALY

## Abstract

The zero-speed fin stabilizer is used to stabilize the roll motion of the ship at full speed. It has two working modes, that is the lift-based normal anti-rolling mode and the drag-based zero-speed anti-rolling mode. Different force generation mechanisms cause different control methods, especially in the form of control. The zero-speed fin stabilizer works in a sinusoidal fashion in normal mode, the same as the conventional fin stabilizer. However, its control form at zero speed is not particularly clear. This paper aims to investigate the control form of fin stabilizer at zero speed through a composite method of theoretical analysis and experimental research. Based on the established reaction force model, the forces generated on the fin flapped in sinusoidal and trapezoidal forms are compared and analyzed. It is found that the trapezoidal form flapping with a small half-cycle ratio generates larger force than the sinusoidal flapping form when the flapping amplitude is the same. The forced rolling tank tests with the fins flapping in sinusoidal and trapezoidal forms were conducted. The test results are consistent with the theoretical analysis results, and the trapezoidal flapping form with a proper small half-cycle ratio is recommended for fin stabilizers at zero speed. The control strategy of fin stabilizer at zero speed is obtained based on the further analysis of the force characteristics of the trapezoidal flapping form and the limitations of the actual fin stabilizer actuation system. The model and full scale roll reduction tests at zero speed were conducted, achieving more than 70% and 60% of the anti-rolling effect respectively. The test results further verify the effectiveness, applicability and practicability of the obtained control form and strategy for fin stabilizers in at-anchor conditions, and can be a reference for engineering practice and other similar studies.

## 1 Introduction

A ship sailing in a seaway inevitably generates six degrees of freedom motions with sea environment disturbance, whether it is in at-anchor, low speed or medium/high speed conditions [1]. Roll motion has the significant impact on ship safety compared with the other five degrees of freedom. Large roll motion can affect the seaworthiness and security of the ship, the reliability and operation of the on-board equipment, the safety of the cargo, the comfort and effectiveness of the crew, and the hit ratio of the weapons for navy vessel [2, 3]. As the roll damping decreases with decreasing sailing speed, the roll motion of ships at zero speed is more severe [4]. With the increase of marine exploitation and utilization, more and more on-board operations need to be performed at low and even zero speed, such as working boat’s lowering and hoisting, on-board helicopter’s taking-off and landing [5, 6]. Therefore, except for the normal speed, it is also necessary to reduce the ship roll motion at zero speed.

The conventional roll reduction devices at full speed are bilge keel, anti-rolling tank, moving weight and gyrostabiliser [7]. The bilge keel is a passive anti-rolling device. It is limited by the sailing resistance and maneuverability, and can not satisfy the stabilization requirement. It is usually used in conjunction with other active anti-roll devices [8]. The anti-rolling tank needs to take up valuable space in the cabin to achieve satisfactory roll reduction effect [9]. The moving weight needs to consume large amounts of power [10]. The gyrostabiliser generates restoring moment through the rotor’s high speed revolution. It is usually mounted in ships of one hundred or two hundred tons for its small roll damping ability [11].

Fin stabilizer is the most effective and most used active roll reduction device [12]. Its anti-rolling effect at high sailing speed can be up to 90% [13]. However, limited by its lift-based force generation mechanism, its roll damping capacity at low and zero speed is poor [14]. In order to solve the problem, the working mode of the fin stabilizer is improved, and a new force generation mechanism, which is explained as “paddle”, is proposed [15–17]. The improved fin stabilizer is called the zero-speed fin stabilizer, as it not only achieves the roll reduction at normal ship speed, but also reduce the ship roll motion at zero speed. Therefore, it has two working modes, that is the lift-based normal anti-rolling mode and the drag-based zero-speed anti-rolling mode [18, 19].

The zero-speed fin stabilizer moves in a sinusoidal fashion according to the wave-induced roll motion in normal anti-rolling mode, just like the conventional fin stabilizers [20]. While its working method in at-anchor conditions is not particularly clear. There are various methods for controlling the fin at zero speed in the existing literature. Dallinga et al. proposed a bang-bang control method, which is to swing the fin from the maximum angle of one side to the maximum angle of the opposite side at each roll cycle [21]. Sebastiani et al. developed the MM-type control method based on multi-variable optimal control theory, which aims to achieve the maximum roll reduction with minimum fin activity [22]. Jin et al. designed a fuzzy controller with minimum energy consumption based on genetic algorithm [23]. Wang proposed a two-step master-slave control law to solve the nonlinear problem between the fin’s movement and the force generated on the fin [24]. Song et al. designed an adaptive controller based on radial basis function and general regression neural network [25]. Su et al. used the fuzzy sliding mode controller to reduce the roll motion at zero speed [26]. Liang et al. achieved the control strategy for zero-speed fin stabilizer based on phase matching between disturbance and compensation in at-anchor conditions [27]. According to the simulation and test results in the above literature, it can be concluded that the control signal of the zero-speed fin stabilizer can be roughly divided into two types: the trapezoidal and sinusoidal fin angle signals. The simulation and test results show that the two forms of control signals have satisfactory anti-rolling effects. However, the pros and cons of these two forms of control signals are not given. This paper aims to investigate the control signal form of zero-speed fin stabilizer in at-anchor conditions through water tank test using a scaled ship model with two pairs of zero-speed fin stabilizers. The structure of this paper is organized as follows. Section 2 establishes the reaction force model generated by a flapping fin at zero speed, and compares and analyzes the reaction forces of sinusoidal and trapezoidal flapping forms. Section 3 details the zero-speed roll reduction control system used for water tank test. Section 4 gives a detailed information of the test results and discussions. Finally, the conclusion is given.

## 2 Theoretical analysis

**Fig 1:**
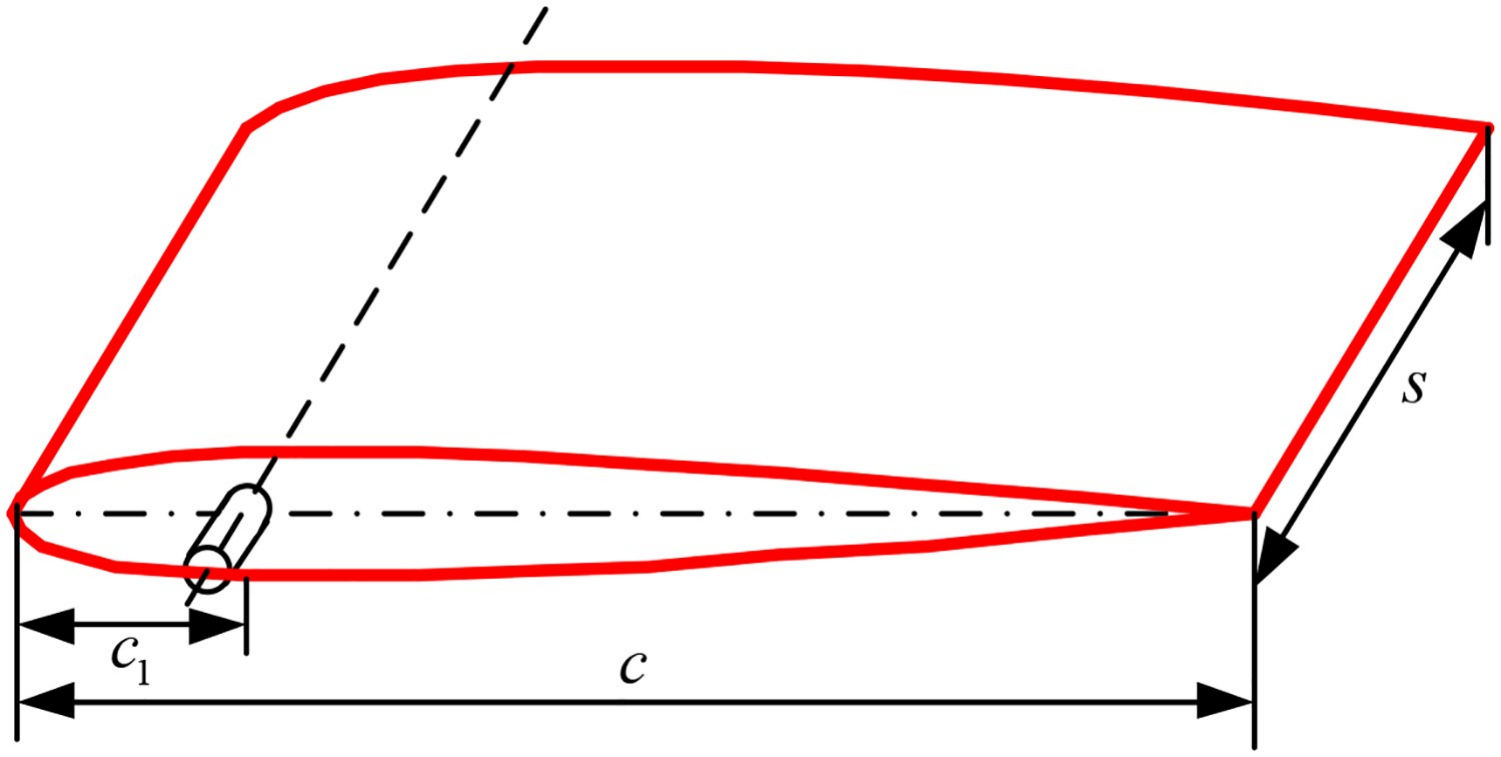
Fin stabilizer and parameter definition.

Just like the oars or paddles of the frog, a reaction force can be generated on the fin at zero speed by active slamming [14]. The force is mainly composed of the pressure drag force, the vortex drag force and added inertia force. To simplify the analysis, the symmetrical fin shown in Fig 1 is selected to model the hydrodynamic forces, where *s*, *c* and *c*_1_ are the span, the chord and the distance between the leading edge and the shaft, respectively.

When the fin is rotated, the fluid discharged by the fin generates a reaction force to the fin that is perpendicular to the fin surface and proportional to the amount of fluid discharged per unit time. The force closely related to the shape and movement of the fin is called as pressure drag, and can be calculated as [20]:

$$\begin{array}{c} F_{pd}=\frac{1}{6}C_{D}\rho s\omega_{f}^{2}(c^{3}-3c^{2}c_{1}+3cc_{1}^{3}) \end{array}$$ (1)

Where *C*_*D*_ is the drag coefficient, *ρ* is the fluid density, *ω*_*f*_ is the fin angular velocity.

The vortex drag is generated from the motion of the fluid in the boundary layer over the fin surface, and can be calculated as [14]:

$$\begin{array}{c} F_{vd}=\frac{1}{6}\rho s\omega_{f}^{2}[3k_{2}{\left(c-c_{1}\right)}^{2}-3k_{2}^{2}(c-c_{1})+(k_{2}^{3}-c_{1}^{3})] \end{array}$$ (2)

Where *k*_2_ is the length of the trailing-edge vortex along the chord direction.

A certain mass of fluid is accelerated or decelerated when the fin is flapped up and downwards, and gives a force to the fin due to their inertia. The force is called as added inertia force and can be calculated as [27]:

$$\begin{array}{c} F_{ad}=\frac{1}{8}k_{a}\pi \rho sc^{2}(c-2c_{1}){\dot{\omega }}_{f} \end{array}$$ (3)

Where *ka* is coefficient related to the fin angular velocity, ${\dot{\omega }}_{f}$ is the fin angular acceleration.

Therefore, the total hydrodynamic force generated on the fin can be expressed as:

$$\begin{array}{c} F=F_{pd}+F_{vd}+F_{ad} \end{array}$$ (4)

It can be seen from the above analysis that the reaction hydrodynamic force generated on the fin at zero speed can be considered to some extent to be composed of two different types of forces, that is the added inertia force proportional to the angular acceleration of the fin and the drag force proportional to the square of the angular velocity of the fin. Taking into account the direction of the force and fin angular velocity, replace $\omega_{f}^{2}$ with *ω*_*f*_|*ω*_*f*_| and rewrite Eq (4) as:

$$\begin{array}{c} F=\rho (K_{1}\omega_{f}|\omega_{f}|+K_{2}{\dot{\omega }}_{f}) \end{array}$$ (5)

Where *K*_1_ and *K*_2_ are the coefficients related to the fin parameters.

It can be seen from Eq (5) that the reaction hydrodynamic force increases with the increase of fin angular velocity and acceleration. Therefore, to achieve better roll reduction effect at zero speed, the force generated on the fin should be as large as possible. However, the fin angular velocity and acceleration are constrained by the physical limitations of the actuation system. Therefore, the force generated on the fin is also limited. When the maximum fin angle is fixed, the way the fin moves determines the angular velocity and acceleration of the fin. Therefore, it is important to determine the optimal control form of fin stabilizer at zero speed for increasing the reaction force and improving the anti-rolling effect. As mentioned above, the control forms in the literature can be roughly divided into sinusoidal and trapezoidal fin angle control. The purpose of this paper is to investigate the optimal control signal form of fin stabilizer in at-anchor conditions.

**Fig 2:**
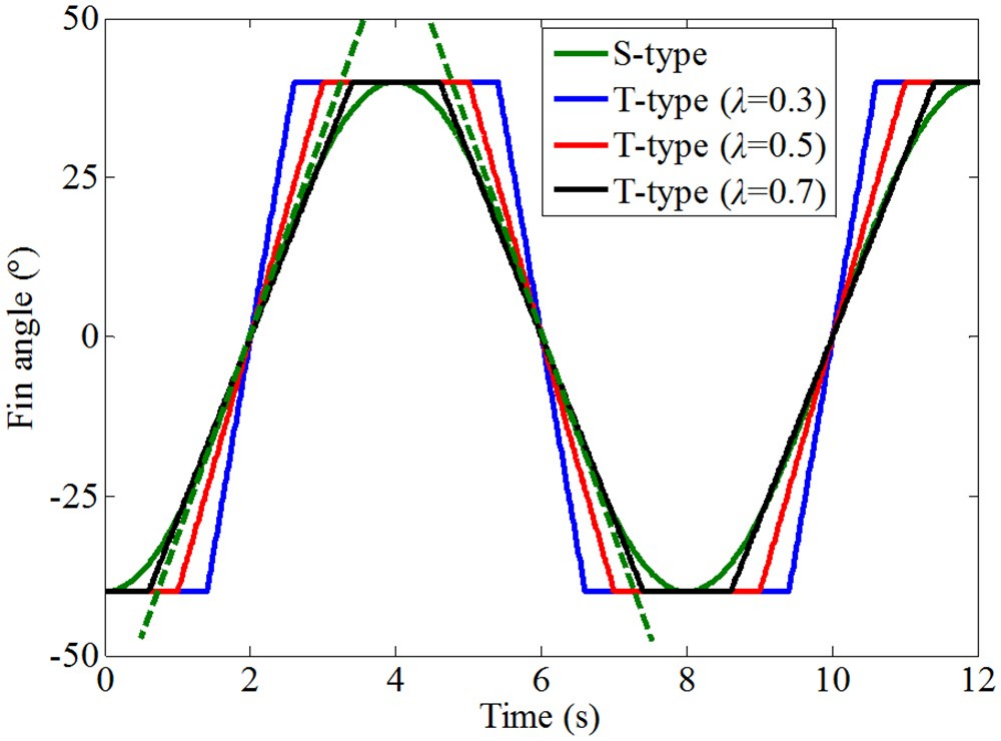
Comparison of sinusoidal and trapezoidal fin flapping forms.

**Fig 3:**
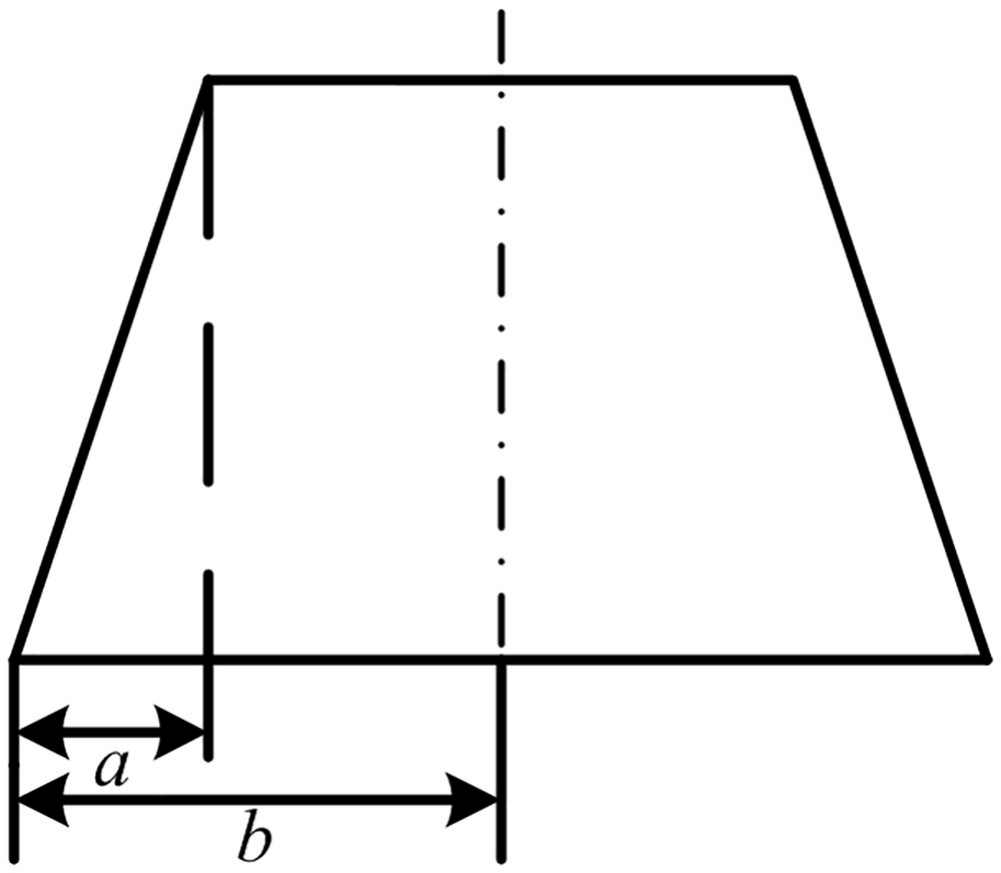
Definition of the half-cycle ratio.

To obtain a large reaction force, the fin is usually flapped from one extreme to the other and back. Without loss of generality, the sinusoidal and trapezoidal fin angles with the maximum fin angle of ±40° and the flapping period of 8 s, marked as S-type and T-type respectively, are show in Fig 2. λ in the legend of Fig 2 represents the half-cycle ratio. As shown in Fig 3, the half-cycle ratio is defined as λ = *a*/*b*. The green dashed line is the tangent of the sinusoidal fin angle signal at the zero-crossing point, corresponding to the half-cycle ratio of 2/*π*.

It should be noted that the trapezoidal fin angle shown in Fig 2 does not actually exist. In practice, the fin angle curve is similar to a trapezoidal type, including an acceleration, a constant-speed transit, a deceleration and a rest. For the convenience of comparison and analysis, the trapezoidal fin angle curve is simplified. During the movement of the fin from -40° to +40° in a sinusoidal form, the angular velocity of the fin increases first and then decreases, and the maximum value is obtained at the zero-crossing point. While the fin angular velocity remains unchanged during the movement in the trapezoidal form. The fin angular velocity moving in trapezoidal form movement with λ < 2/*π* is larger than that moving in sinusoidal form during fin motion. Larger fin angular velocity means larger hydrodynamic reaction force.

**Fig 4:**
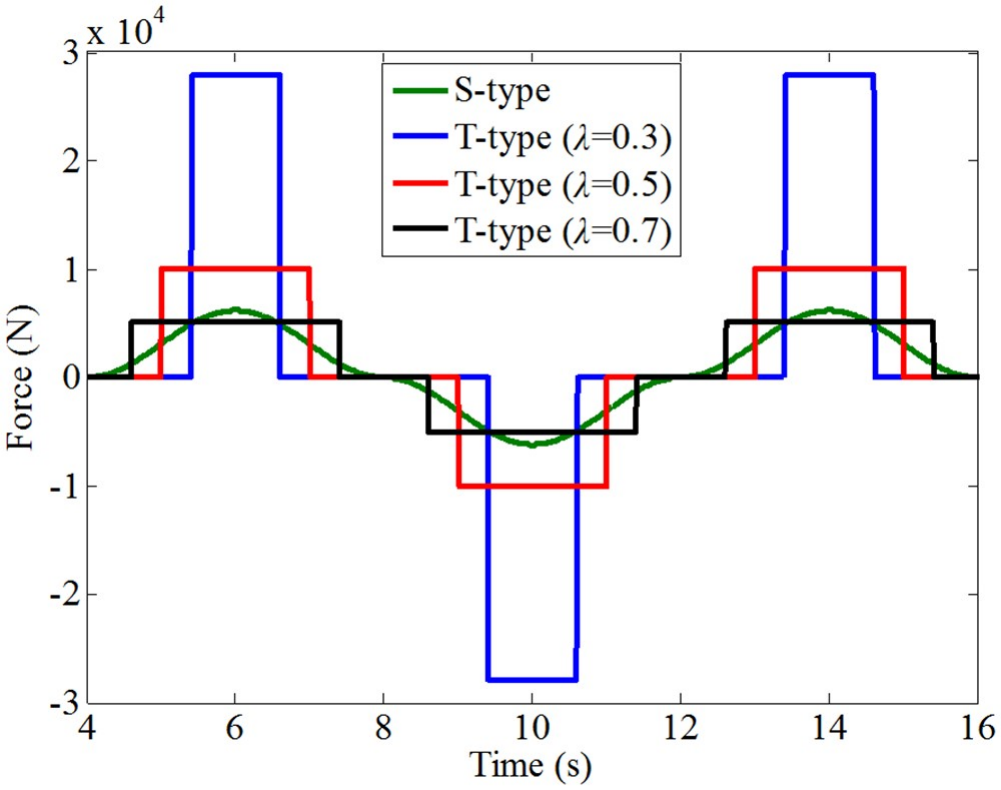
Comparison of the forces generated on the fin with different flapping forms.

As any acceleration phase at the kick-off of the fins stroke has always to be followed by an opposite deceleration phase at the end of the stroke, the two inertia contributions associated with the angular acceleration of the fin more or less cancel each other. For analysis convenience, neglecting the added inertia force, the drag forces of the two control forms are given, as shown in Fig 4. The forces are calculated using Eq (5) with *ρ* = 1000 kg/m^3^, *K*_1_ = 20.58 and *K*_2_ = 4.974 from [28]. The amplitude and duration of the force are given in Table 1. It can be seen that the amplitude of the reaction force increases and and duration decreases with a decrease in the half-cycle ratio, respectively. Although the reaction force generated on the fin moved in sinusoidal form exists throughout the motion, its amplitude is only large near the zero-crossing point of the fin angle. In general, the reaction force generated on the fin flapped in trapezoidal form with half-cycle ratio less than 2/*π* is greater than that in sinusoidal form, which means the fin has a greater anti-rolling effect when it is flapped in a trapezoidal form with proper half-cycle ratio at zero speed. However, small half-cycle ratio not only requires large power, but also leads to unpleasant jerk. Therefore, therefore, it is important to select a proper half-cycle ratio within the limitation of actuation system. Further verification analysis is carried out by water tank test given in Section 4.

**Table 1:** Amplitudes and durations of the reaction forces in different flapping forms.

Flapping form	S-type	T-type (λ = 0.3)	T-type (λ = 0.5)	T-type (λ = 0.7)
Force amplitude (KN)	3.09 (average value)	27.86	10.03	5.12
Force duration (s)	4	1.2	2	2.8

## 3 System description

**Fig 5:**
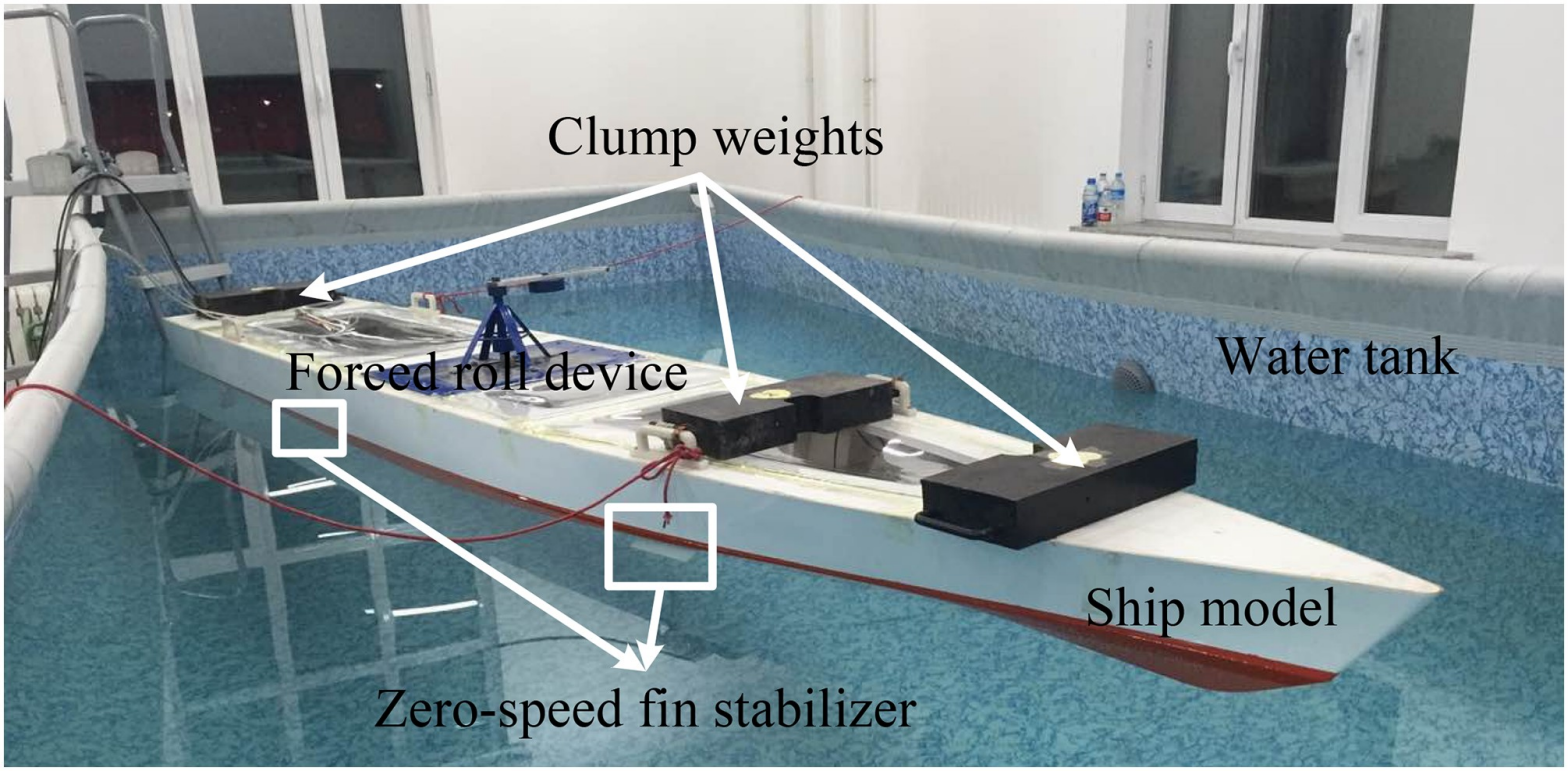
Zero-speed roll stabilization control system.

The zero-speed roll stabilization control system, as shown in Fig 5, is designed and built by the Institute of Ship Stabilization and Control Research of Harbin Engineering University. The whole system is composed of a water tank, a scaled ship model, an attitude sensor module, two pairs of fin stabilizer models and their electric drive steering gears, a forced roll system and its drive system, several clump weights and two PCs (one is used to run the drive control program of the forced roll device, and the other is used for fin control and data acquisition).

### 3.1 Ship model

An 84-meter-long fishery ship is selected as the research object. The scaled model of the fishery ship, with the scaled ratio of 1:25, is built to conduct the water tank test. The main parameters of the ship and the scaled model are shown in Table 2.

**Table 2:** Main parameters of the ship.

Description	Symbol	Prototype	Model
Length between perpendiculars (m)	*L*	84	3.36
Beam over all (m)	*B*	10	0.4
Draft (m)	*d*	3.2	0.128
Displacement (t)	*D*	1300	0.0832
Transverse metacentric height (m)	*h*	1.1	0.04
Roll period (s)	*T*_*φ*_	8.5	1.7

### 3.2 Fin model

As shown in Fig 5, the reduce-scaled ship model is equipped with two pairs of parallelogram fin stabilizers. As mentioned in Section 2, the hydrodynamic force generated on a fin using “paddle” mode mainly consists of the pressure drag, vortex drag and added mass force [14, 19, 20, 27]. The pressure drag and vortex drag are proportional to the square of the fin angular velocity, and the added mass force is proportional to the fin angular acceleration [22, 28–30]. Therefore, when the fin’s movement is determined, the hydrodynamic force generated on the fin can be increased by optimize the fin shape parameters. According to the simulation and test results of Jin et al. [20] and Song et al. [31], the shaft position, aspect ratio and trailing shape have the most significant influence on the hydrodynamic force when the fin area is constant.

#### 3.2.1 Fin shaft position

Affected by the special working mode at zero speed, the hydrodynamic forces generated on the front and rear sides of the fin shaft have the opposite direction. The force generated on the front side of the fin shaft acts to reduce the total anti-rolling force. Therefore, it is a effective way to move the fin shaft forward to increase the force generated at zero speed. However, the forward movement of the fin shaft will greatly increase the driving torque at medium and high speeds. Therefore, the fin shaft position in practical applications shall be determined based on the specific ship type, operation conditions and anti-rolling indicators, and the recommended fin shaft position is 1/6∼1/5 of the fin chord from the leading edge [31].

#### 3.2.2 Aspect ratio

When the area and shaft position of the fin are determined, the smaller the aspect ratio, the larger the area on the rear side of the fin shaft, and therefore the greater the hydrodynamic force generated on the flapping fin. However, a smaller aspect ratio is not conducive to the roll reduction of the ship at medium and high speeds. Therefore, the determination of the aspect ratio of the zero-speed in stabilizer needs to be considered comprehensively based on actual conditions, and the recommended aspect ratio is 0.35∼0.6 [20].

#### 3.2.3 Parallelogram fin

It can be seen from the above analysis that the purpose of changing the fin shaft position and the aspect ratio is to increase the generated effective damping force by increasing the area of the rear side of the fin shaft. However, both the changes of the fin shaft position and aspect ratio have contradictions in the applications of zero/low speed and medium/high speed. One way to resolve this contradiction is to change the conventional trapezoidal or rectangular fin to the parallelogram fin. In the case where the fin area, shaft position and aspect ratio are fixed, the increased trailing edge area on the rear side of the fin shaft can greatly increase the anti-rolling force; meanwhile, the decrease of the leading edge area on the front side of the fin shaft reduce the loss of the effective anti-rolling force. Moreover, the change has little effect on the driving torque at medium and high speed, and increases the lift coefficient at medium and high speeds. The inner acute angle of the parallelogram fin can be used to describe the change, and the recommended value is 60°∼80° [31].

#### 3.2.4 Fin parameters

**Fig 6:**
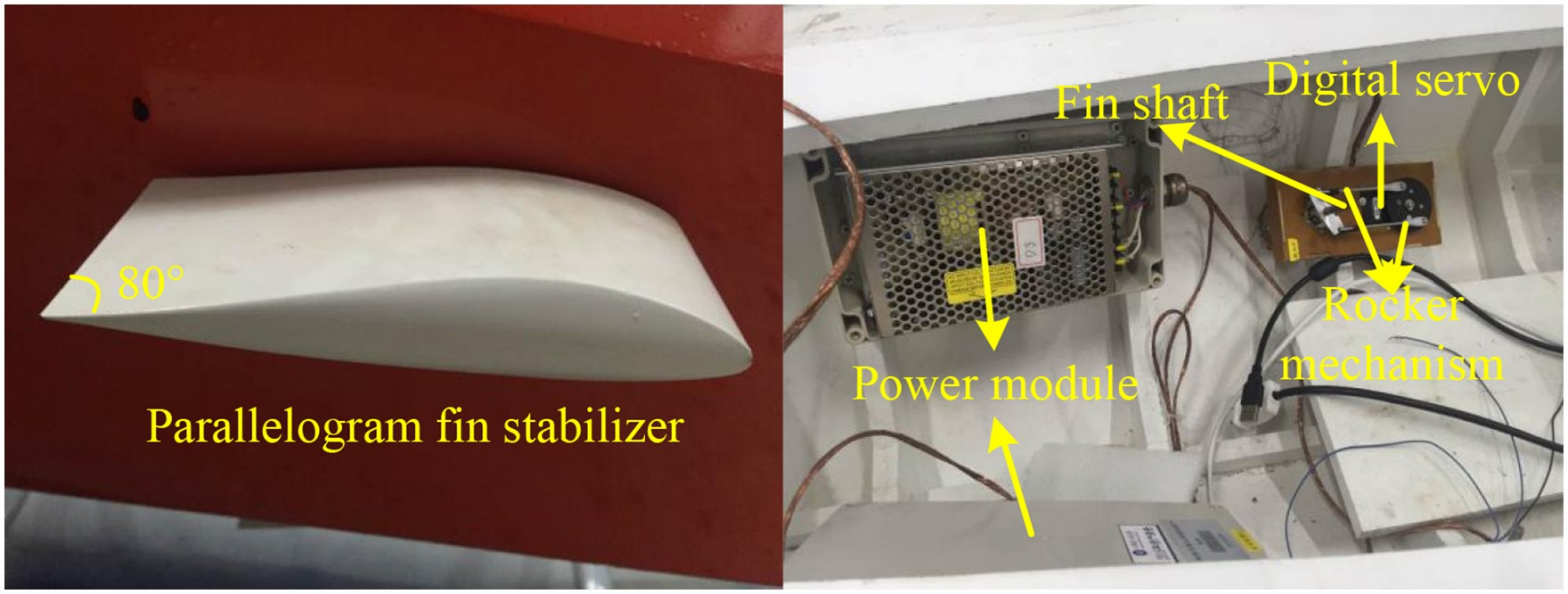
Parallelogram fin stabilizer model and its driver module.

Based on the above analysis, two pairs of parallelogram fin stabilizers are designed to satisfy the roll reduction requirement at full speed. The main parameters of the fin and fin model are shown in Table 3. The distance between the fin shaft and the leading edge of the fin is 1/5 of the chord. The inner acute angle is 80°. Fin stabilizer model and its driver module are shown in Fig 6. The fins are driven by the digital servo Dynamixel MX-64 of BJROBOT. The standstill torque and no-load speed at 12 V operating voltage are 6.0 Nm and 63 rpm, respectively. It should be noted that in order to explore the effect of trapezoidal flapping form with different flapping amplitudes and half-cycle ratios, the maximum flapping amplitude of the fin stabilizer model is set to ±60°, and the fin rate of the model is not limited.

**Table 3:** Main parameters of the fins.

Description	Symbol	Prototype	Model
Fin area (m^2^)	*A*	3.92	0.006272
Aspect ratio	Λ	0.5	0.5
Chord (m)	*c*	2.8	0.112
Span (m)	*s*	1.4	0.056
Maximum fin angle (°)	*α*_*max*_	±40	±60
Maximum fin rate (°/s)	${\dot{\alpha }}_{max}$	50	-
Roll arm (m)	*l*_*f*_	5.7	0.228

### 3.3 Forced roll system

**Fig 7:**
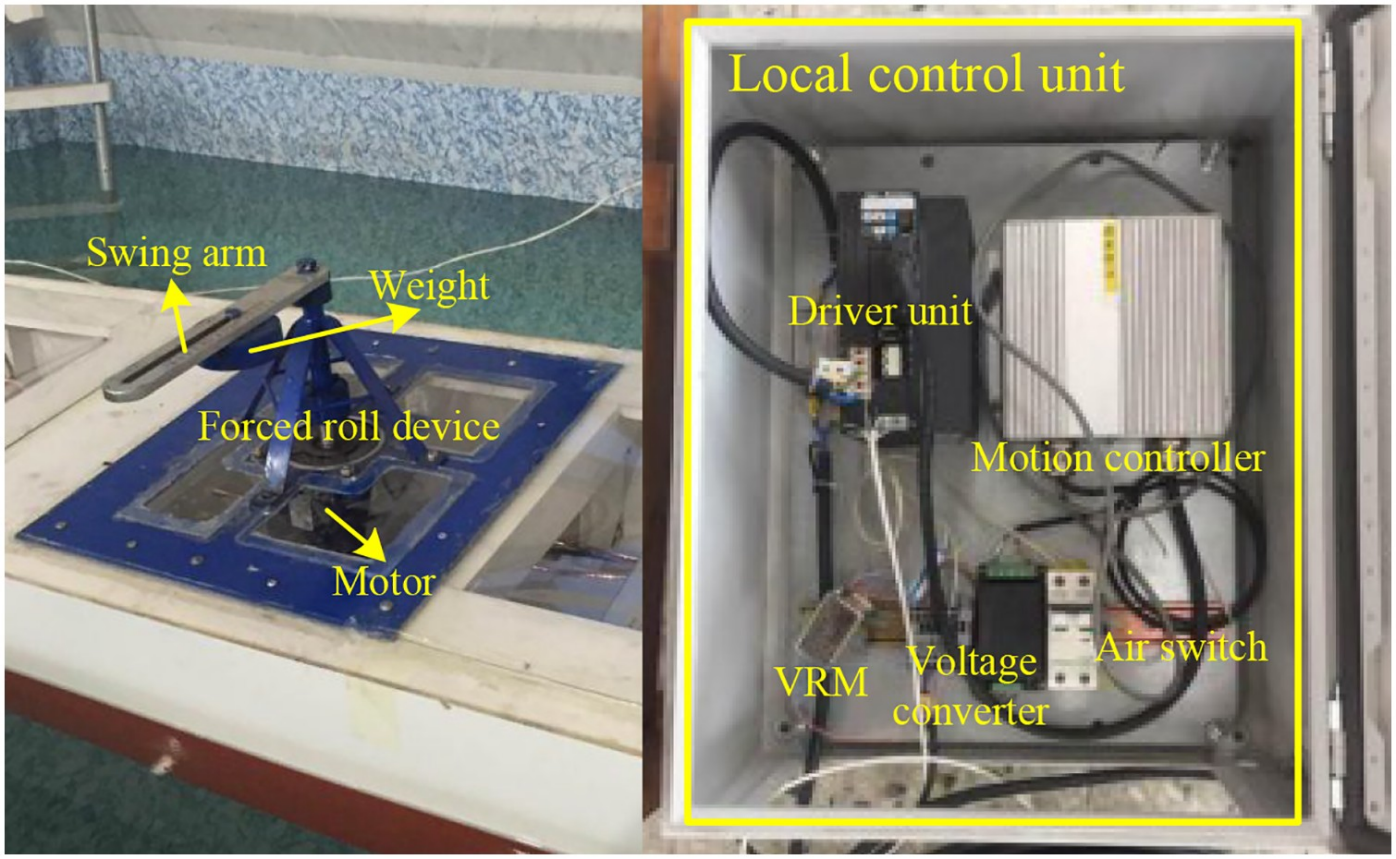
Forced roll device and its local control unit.

As shown in Fig 7, the forced roll system is used to force the ship model to roll around its longitudinal axis according to the command signal. The forced roll system consists of a control PC, a driving motor, a local control unit and a forced roll device. The frequency and amplitude of the forced roll motion of the ship model can be changed by adjusting the rotation speed of the forced roll device and the position of the weight on the swing arm, respectively. For safety considerations, the weight is fixed to the middle of the swing arm to limit the amplitude of the forced roll amplitude. The driving motor of the forced roll system is the S-FLAG series of Sankyo, and the maximum rotation speed corresponding to the input voltage of 10 V can be up to 3000 rpm.

### 3.4 Counterweight

The relationship between the ship model and the real ship shall satisfy not only the geometric similarity and mass distribution similarity, but also the similarity of the center of gravity [32]. Therefore, the ship model shall be counter-weighted using clump weights to meet the principle of similarity. The suspension method is used to carry out the counterweight of the ship model. There are four clump weights used here. Three of them are on the deck and the forth one is fixed in the cabin behind the forced roll device. The state of the ship model after counterweight is shown in Fig 5.

### 3.5 PC program

**Fig 8:**
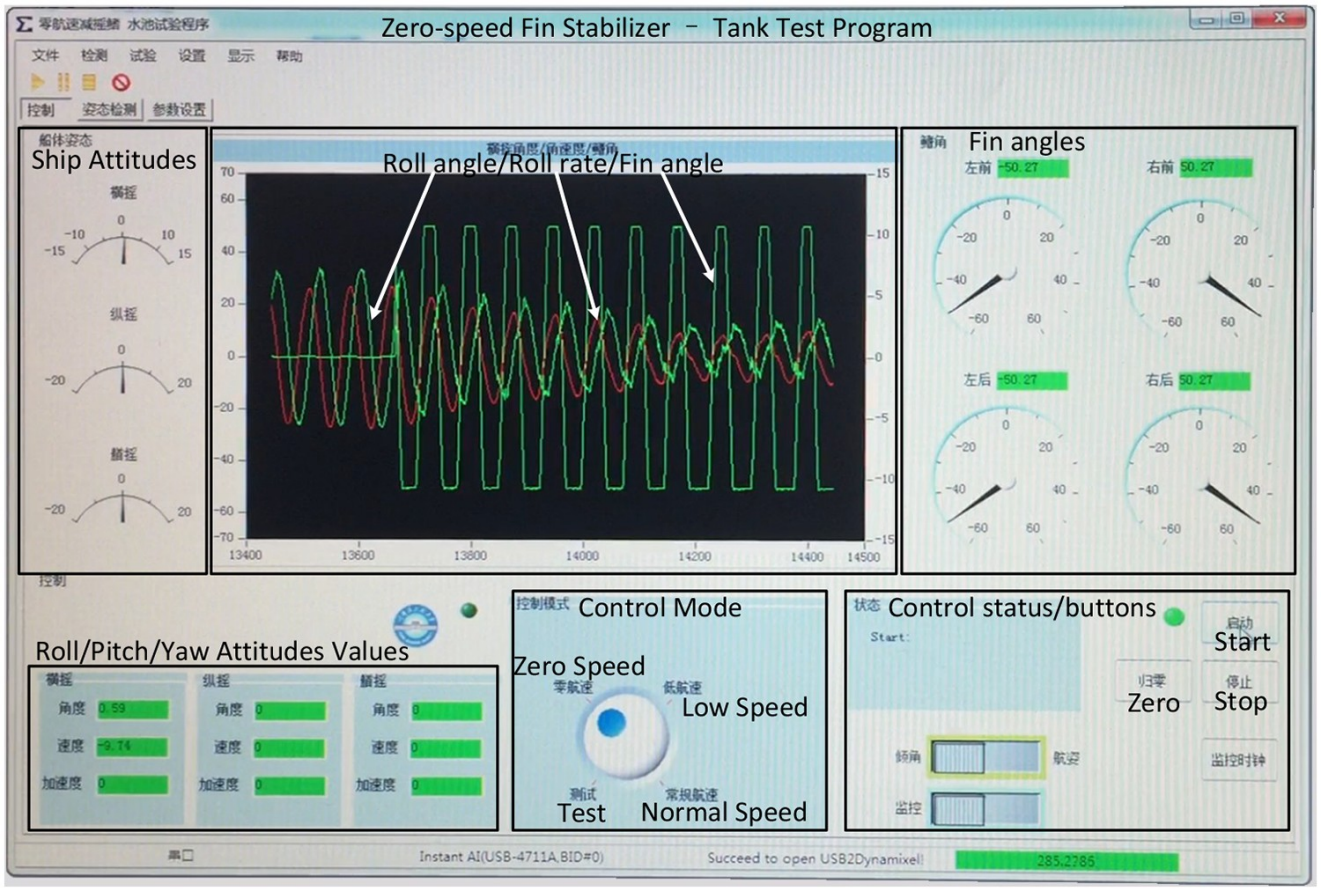
The host computer interface.

The man-machine interface of the PC program, as shown in Fig 8 is written by C#. The main functions of the PC program are data acquisition, display and storage, control mode selection, control parameter configuration and so on. The PC program runs on a computer with an Intel Core i5 650@3.2GHz CPU and 4 GB of RAM, running a 64-bit Win 7.

## 4 Experiments and discussions

### 4.1 Free roll decay test

**Fig 9:**
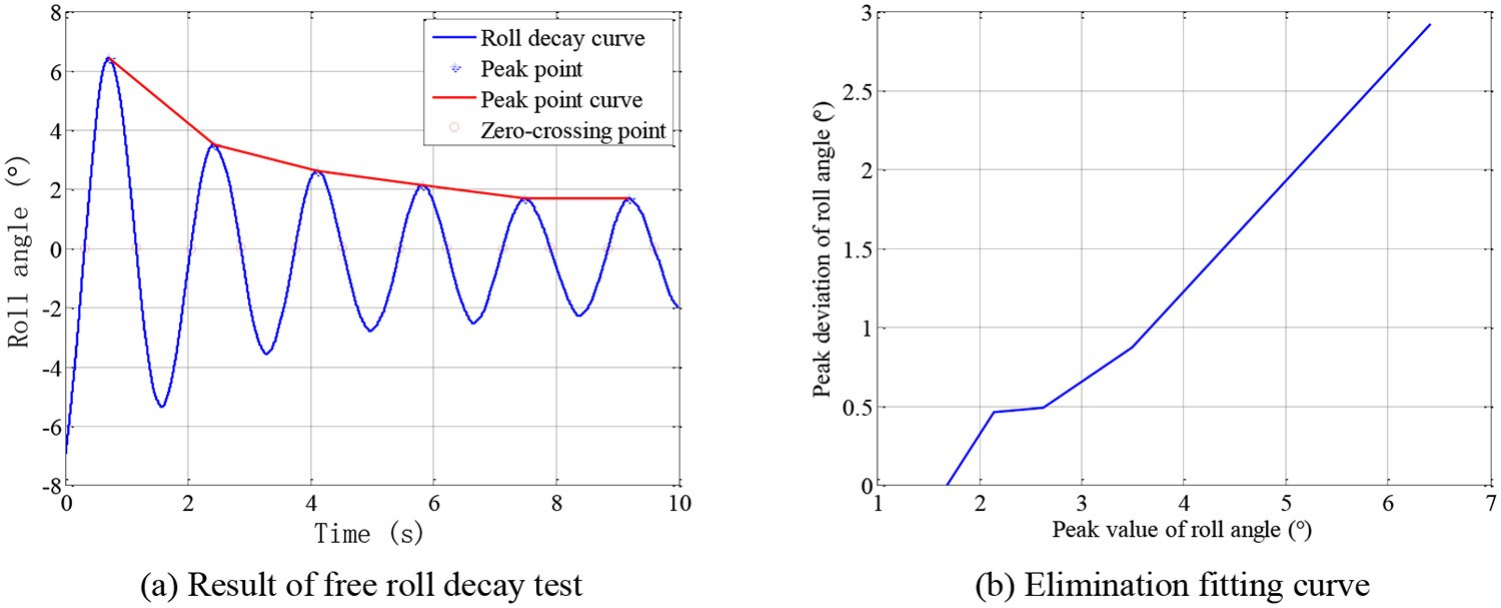
Roll decay curve and its elimination fitting curve.

The free roll decay test is carried out to obtain the natural rolling period and dimensionless damping coefficient of the scaled ship model. Place the ship model into the water and apply a downward force on one side of the ship model to tilt it to a certain heel angle. Then remove the force quickly after the water surface is calm. The ship model rolls around its longitudinal axis under the action of the recovering moment and inertia moment. The amplitude of the roll motion is gradually reduced to zero under the damping effect of water. The roll decay motion is measured by the roll rate sensor, and recorded and stored by the PC program. A total of 8 sets of free roll decay tests were performed. A typical set of roll decay curve and its elimination fitting curve are shown in Fig 9. The results of the 8 sets of free roll decay tests are shown in Table 4, where *T*_*P*_ is the peak-to-peak period, *T*_*Z*_ is the cross-zero period and *n*_*u*_ is the dimensionless decay coefficient.

**Table 4:** Roll period and damping coefficient obtained in free roll decay test.

No.	1	2	3	4	5	6	7	8	Average
*T*_*P*_ (s)	1.6997	1.6954	1.7060	1.6998	1.7116	1.6966	1.7020	1.7052	1.7020
*T*_*Z*_ (s)	1.6835	1.6847	1.6936	1.6865	1.7004	1.6880	1.6929	1.6962	1.6907
*n*_*u*_	0.1594	0.1580	0.1562	0.1748	0.1949	0.1494	0.1734	0.1599	0.1658

Due to the size of the water tank, the roll decay motion is disturbed by the wall echo. It can be seen from Fig 9 that the roll decay motion after 4 periods experiences larger disturbance. Therefore, the calculation of the roll period and damping coefficient is accomplished using the data of the first four periods. The natural rolling period of the ship model, 1.6964 s, is obtained by averaging *T*_*P*_ and *T*_*Z*_. It can be seen from Table 1 that the obtained natural rolling period of the ship model is almost the same as the theoretical value, 1.7 s. The dimensionless damping coefficient, 0.1658, is obtained by averaging *n*_*u*1_∼*n*_*u*8_.

### 4.2 Forced rolling test with fin stabilizer only

In order to explore the anti-rolling potential of the designed parallelogram fin stabilizer under different types of fin angle control forms, the forced rolling tests with fin stabilizer working in a sinusoidal and trapezoidal manner were implemented. The flapping period is set to 1.6964 s, which is the same as the natural rolling period of the ship model.

#### 4.2.1 Forced rolling test with sinusoidal fin angle signal

**Fig 10:**
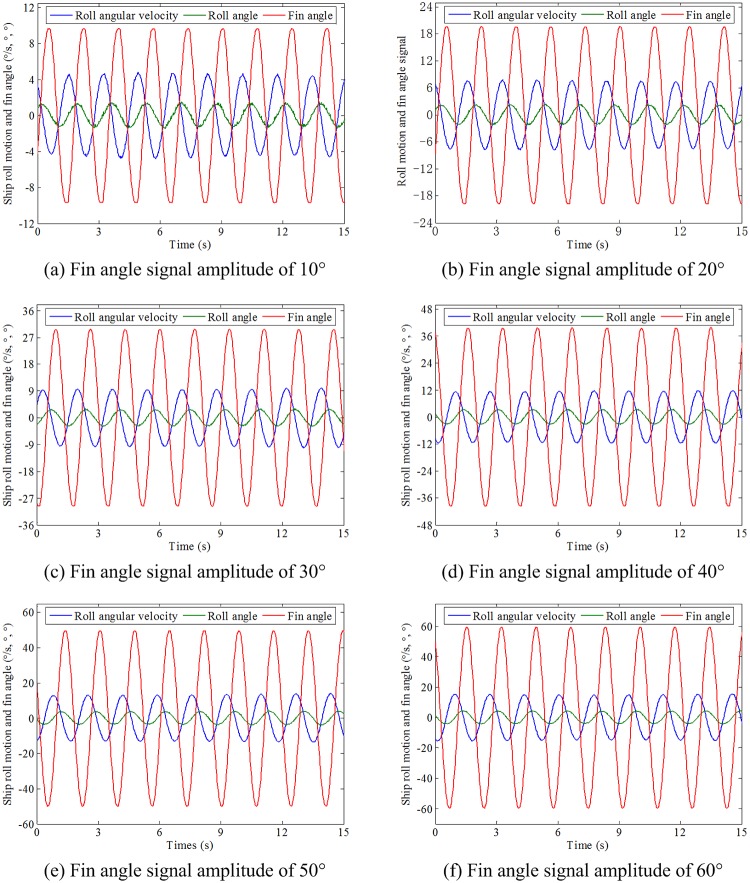
Roll motion under different amplitude sinusoidal fin angle control signals.

The amplitude of the sinusoidal fin angle signal ranges from 10° to 60° with the interval of 10°. The results of the forced rolling test with sine-based fin angle control signal of different amplitudes are shown in Fig 10.

To have a visible comparison, the maximum forced roll angles under different amplitude sinusoidal fin angle control signals are shown in Table 5. It can be seen from Table 5 that the maximum forced roll angle increases with the increase of the amplitude of the fin angle signal. The maximum forced roll angle can be up to 4.17° with the fin angle signal amplitude of 60°.

**Table 5:** Ship roll motion under different amplitude sinusoidal fin angle signals.

Description	Value					
Fin angle signal amplitude (°)	10	20	30	40	50	60
Maximum forced roll angle (°)	0.97	1.98	2.70	3.18	3.67	4.17

#### 4.2.2 Forced rolling test with trapezoidal fin angle signal

**Fig 11:**
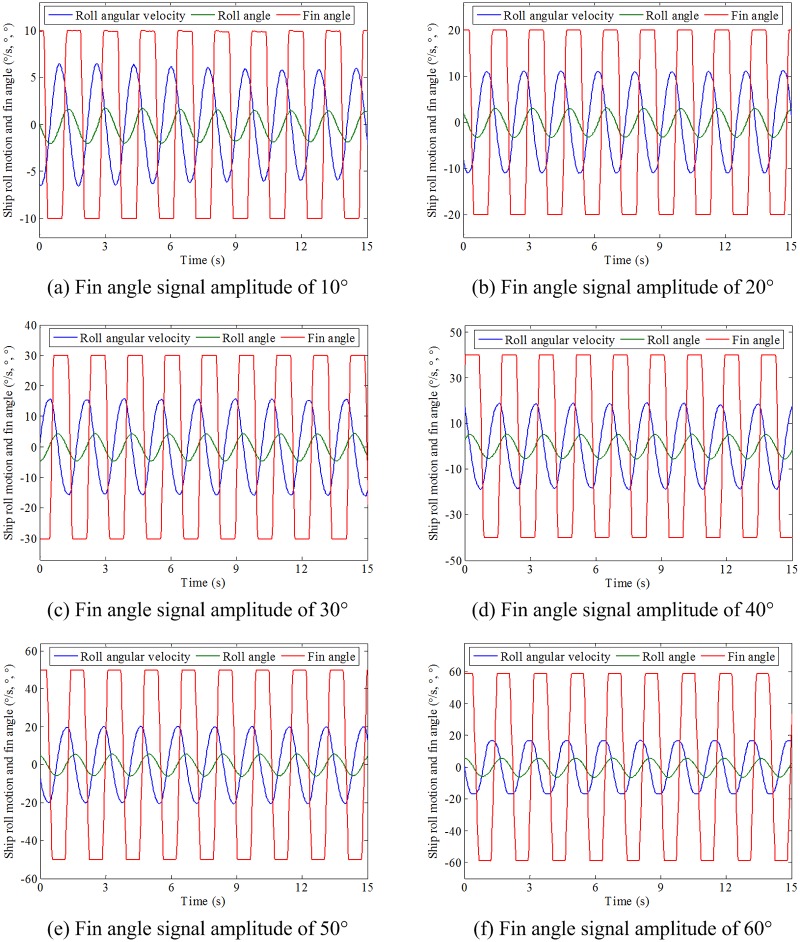
Forced roll motion with half-cycle ratio of 0.2 and different signal amplitudes.

The amplitude of the trapezoidal fin angle signal is set to 10°∼60° with the interval of 10°. In order to explore effect of half-cycle ratio of the trapezoidal fin angle signal on the roll motion, the half-cycle ratio is set to 0∼0.5 with the interval of 0.1. Therefore, a total of 36 sets of tests with different signal amplitude and half-cycle ratio combinations were carried out. The results of the forced rolling test with trapezoidal fin angle signal are shown in Table 6. Limited by the length, the results of the forced rolling test with half-cycle ratio of 0.2 and different signal amplitudes are shown in Fig 11.

**Table 6:** Ship roll motion under different amplitude trapezoidal fin angle control signals.

Fin angle signal amplitude (°)		10	20	30	40	50	60
**Maximum forced roll angle (°)**	λ = 0.0	3.10	3.73	4.63	5.28	5.85	5.92
	λ = 0.1	2.12	3.56	4.46	5.23	5.84	5.91
	λ = 0.2	1.74	3.18	4.45	5.22	5.83	5.90
	λ = 0.3	1.12	2.54	3.62	4.63	5.50	5.82
	λ = 0.4	1.05	2.10	3.20	4.01	4.84	5.32
	λ = 0.5	1.01	2.02	2.92	3.64	4.34	4.80

#### 4.2.3 Discussion and analysis

**Fig 12:**
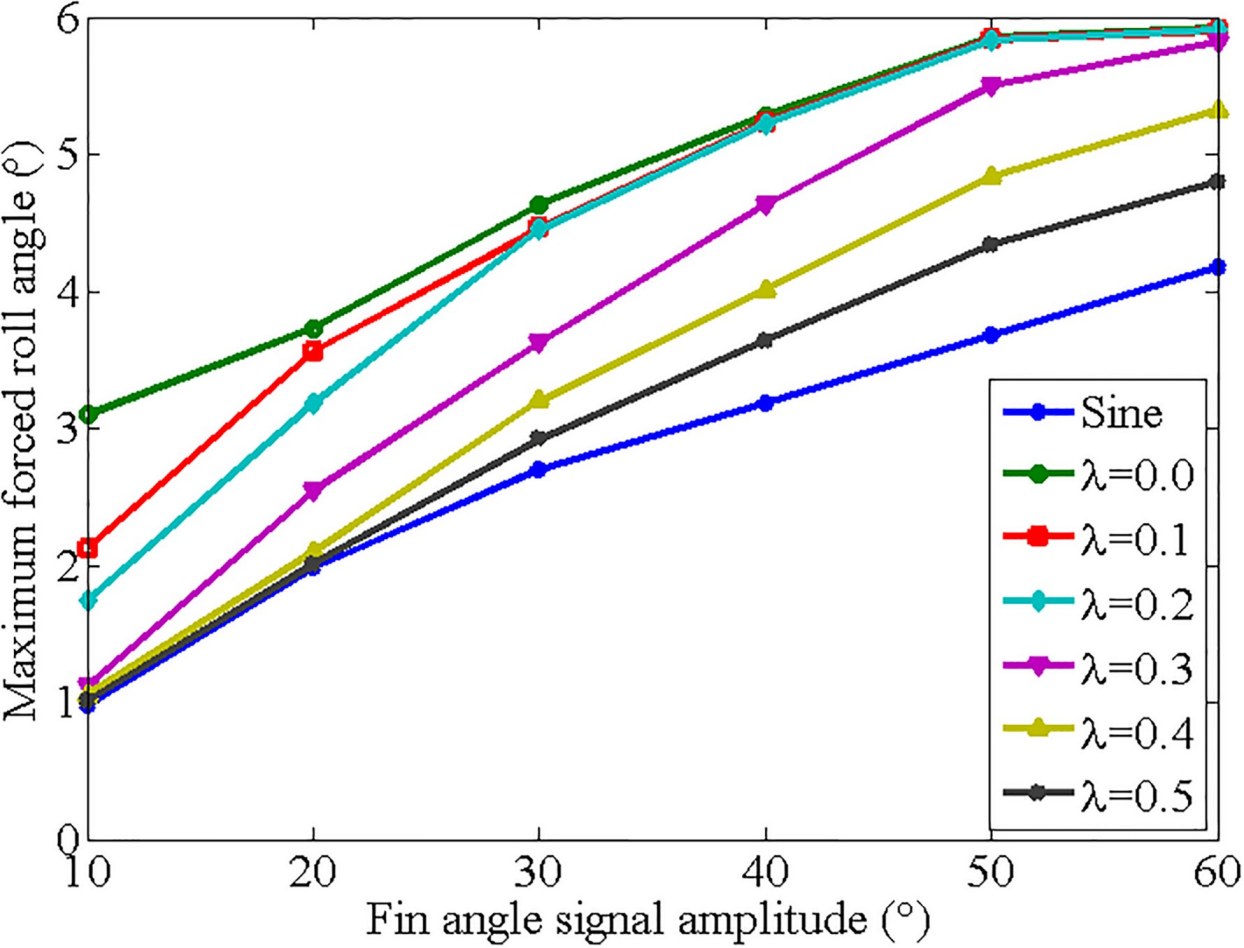
Forced roll motion with different types of fin angle signals.

For comparison purpose, the test results of the forced rolling test with sinusoidal and trapezoidal fin angle signals are summarized, as shown in Fig 12. It can be seen from Fig 12 that the maximum forced roll angle increases with the increase of fin angle signal amplitude when the half-cycle ratio is constant. When the fin angle signal amplitude is fixed, the forced roll angle substantially decreases as the half cycle ratio increases. It can also be seen that the forced roll angles are almost the same when the fin angle amplitude is large than 30° and the half-cycle ratio is less than 0.2. This is mainly due to the fact that the required fin rate exceeds the limit of the drive servo. When the fin angle amplitude is less than 20° and the half-cycle ratio is greater than 0.3, the resulting forced roll angles are also relatively close. The small fin angle amplitude limits the availability of maximum fin rate and the generated reaction force to roll the ship model. It can be concluded from the overall trend of the results that at the same flapping amplitude, the forced roll angle with the fins flapped in trapezoidal form with a proper small half-cycle ratio is larger than that moved in sinusoidal form, which verifies the results obtained by theoretical analysis in Section 2. Therefore, it is recommended to flap the fins in a trapezoidal form at zero speed with a small half-cycle ratio and a large flapping amplitude to generate a large reaction force to improve the anti-rolling effect. It seems that the following two zero-speed roll reduction control strategies can be obtained by analyzing the results of the forced rolling test:

1. Fix the half-cycle ratio, and change the fin’s flapping amplitude according to the ship’s roll information;
2. Fix the fin’s flapping amplitude, and change the half-cycle ratio according to the ship’s roll information.

However, it should be noted that the forced rolling test does not limit the maximum fin angle and fin rate according to the actual system for exploration purpose. The above two methods are essentially the same after considering the actual maximum fin angle and fin rate limit. When the maximum fin angle and fin rate are fixed, the minimum half-cycle ratio is also determined according to the definition of the half-cycle ratio. The maximum reaction force generated by the flapping fins can also be determined. Therefore, the anti-rolling effect of fin stabilizers at zero speed decreases as the sea conditions increase, which is the same as that obtained in the literature.

According to Conolly [33], the roll motion of the ship equipped with fin stabilizers can be expressed by the following linear equation as:

$$\begin{array}{c} (I_{x}+\Delta I_{x})\ddot{\phi }+2N_{u}\dot{\phi }+Dh\phi =K_{W}+K_{C} \end{array}$$ (6)

Where *ϕ* is the roll angle, *I*_*x*_ and Δ*I*_*x*_ are the inertia moment and added inertia moment, respectively, 2*N*_*u*_ is the roll damping torque per unit rolling angular velocity, *D* is the ship’s displacement, *h* is the transverse metacentric height, *K*_*W*_ is the wave disturbance moment and *K*_*C*_ is the anti-rolling control moment generated by the fins.

**Fig 13:**
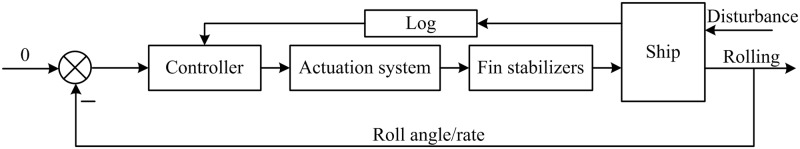
Diagram of roll reduction principle.

It can be seen from Eq (6) that the ship will stop rolling if the wave disturbance moment is fully compensated by the fin-induced anti-rolling control moment. Although the forced generation mechanism at zero speed is different from that with speed, it is very effective to reduce the roll motion of the ship by increasing the roll damping for both cases. A simple way to increase the equivalent roll damping is to generated a large reaction force at the moment the roll velocity is highest. Based on the analysis of the characteristics of the reaction force generated on the fin flapped in a trapezoidal form, the “bang-bang” control method is adopted. That is to control the fins to move fast from one extreme position to the other when the ship’s roll angular velocity is largest. It is known that the roll velocity achieves the highest value at the zero-crossing point of the roll angle. Therefore, the fin angle command can be obtained based on the feed-back from roll angle and roll rate in order to reduce the roll motion of the ship. As shown in Fig 13, the roll reduction control principle at zero speed is similar to that with speed.

When the roll period and the maximum fin angle are fixed, the half-cycle ratio is inversely proportional to the fin rate according to its definition. Therefore, in this case the minimum half-cycle ratio is achieved when the fin is flapped at the maximum fin rate. In order to increase the effectivity of zero-speed operation, the fin stabilizers should be flapped in their maximum fin angles and minimum half-cycle ratio to generate a largest reaction force. This is true for a large roll motion. However, due to the randomness of the waves, the ship has a small rolling amplitude at some point. If the fins continue to be controlled to flapped at the maximum amplitude and the minimum half-cycle ratio, there will be a phenomenon of over anti-rolling. In this case, it may be practical and economic to reduce the fin’s flapping amplitude, which is equivalent to increasing the half-cycle ratio while ensuring the fastness of the fin flapping. The following formula can be used to calculate the flapping amplitude of the fins as:

$$\begin{array}{c} \alpha_{\max}^{*}=\alpha_{0}+K(\alpha_{\max}-\alpha_{0}) \end{array}$$ (7)

**Fig 14:**
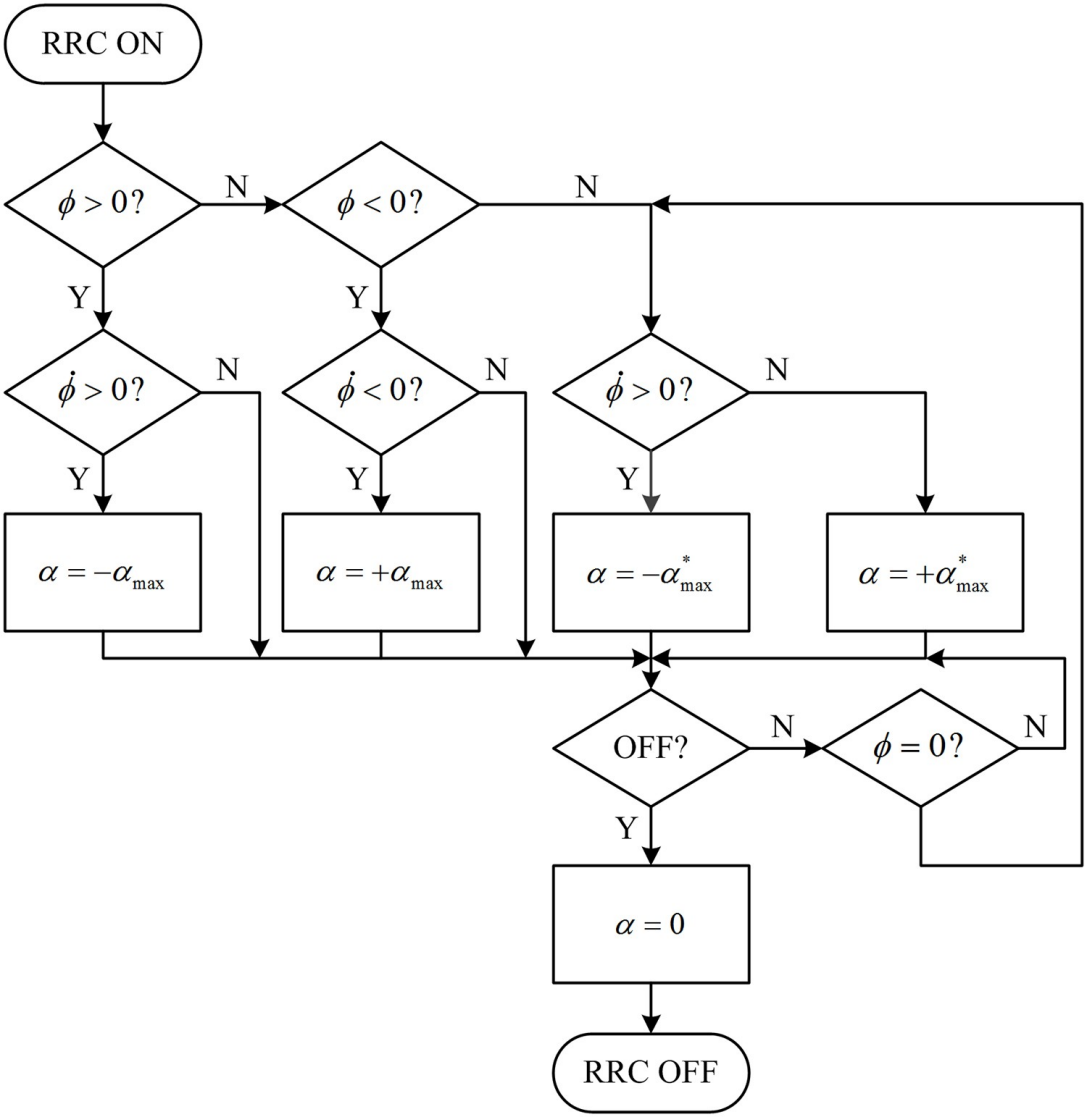
Diagram of the control logic for RRC at zero speed.

Where $\alpha_{\max}^{*}$ is the fin’s flapping amplitude, *α*_max_ is the max fin angle, *α*_0_ is the lower limit of the flapping amplitude to ensure the effectivity of zero-speed operation, K is the weighting coefficient that determines the amplitude of the fin flapping, and its value is between 0 and 1 depending on the magnitude of the ship’s roll motion. The Kalman filter is adopted here to predict the next roll peak to calculate the weighting coefficient based on the roll rate at the zero-crossing point of the roll angle. Based on the analysis above, the following control logic can be obtained, as shown in Fig 14. RRC in Fig 14 means roll reduction control. The roll rate when the ship is rolling to the port side is defined as the positive roll rate. The final position of the fin’s flapping to roll the ship to the port side is defined as positive. The fins should be fast flapped to the positive or negative extreme position to prepare for the next anti-rolling flapping according to the rolling information when the RRC is turned on.

### 4.3 Roll reduction test

#### 4.3.1 Model tank test

**Fig 15:**
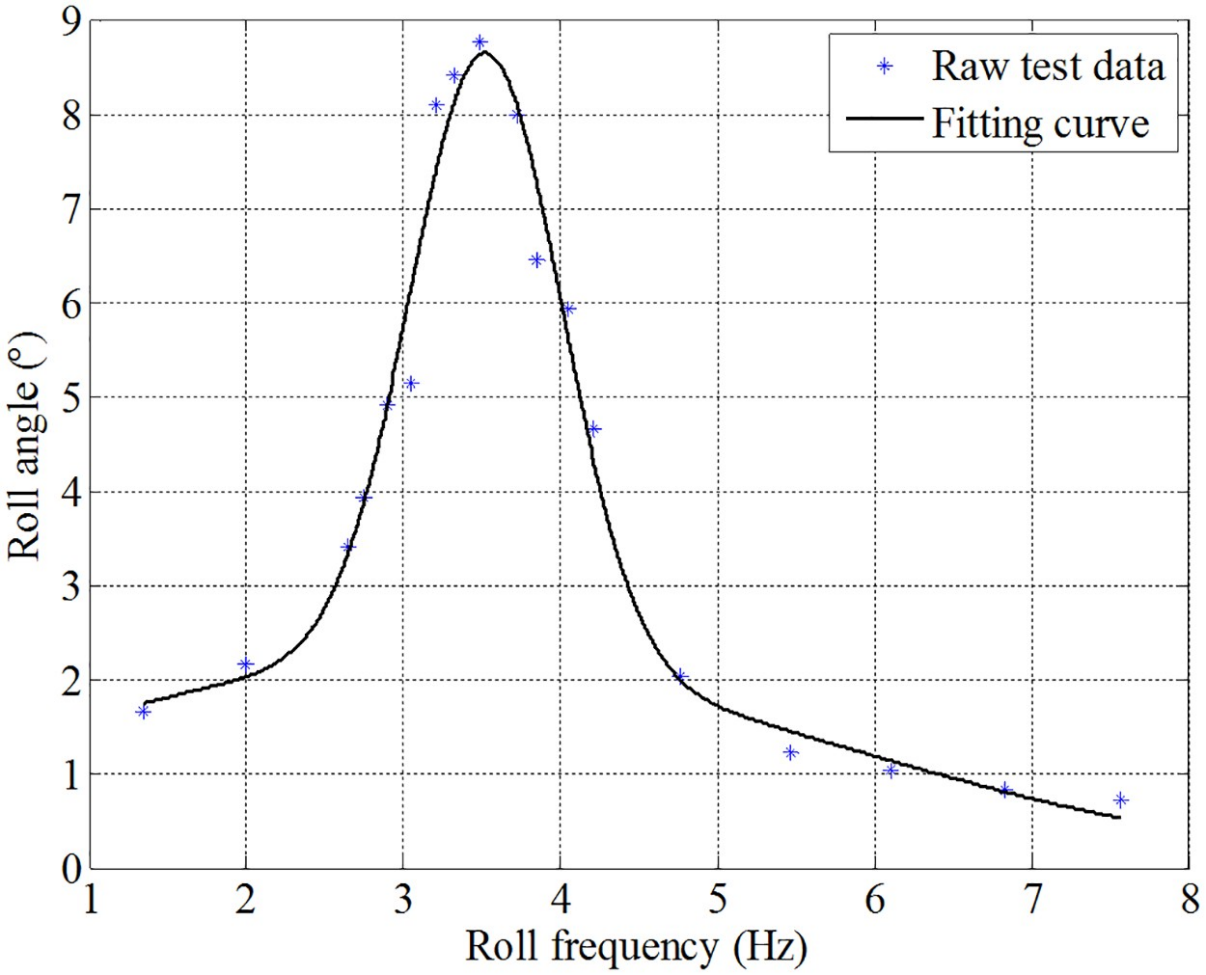
Amplitude-frequency characteristics of the roll motion of the ship model.

Roll reduction model tank test was conducted to investigate the effect of fin stabilizers at zero speed under the control strategy obtained in Section 4.2.3. The forced rolling device is used to generate the roll disturbance moment to roll the ship model. The amplitude-frequency characteristics of the roll motion of the ship model under the action of the forced rolling device is shown in Fig 15. It can be seen that the maximum roll response occurs at the roll frequency of about 3.7 Hz. The corresponding roll period can be easily calculated as 2*π*/3.7 = 1.698 s, which further validates the results of the free roll decay test. The model roll reduction tank test was carried out at this frequency.

**Fig 16:**
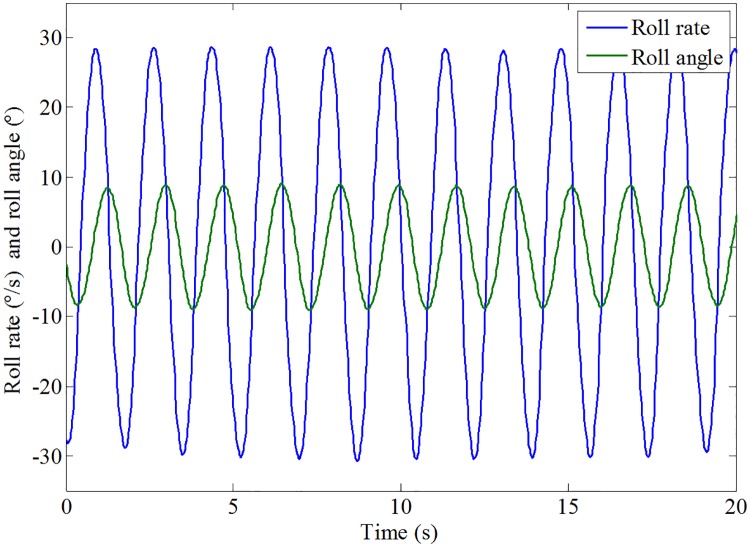
Roll response of the ship model without RRC.

**Fig 17:**
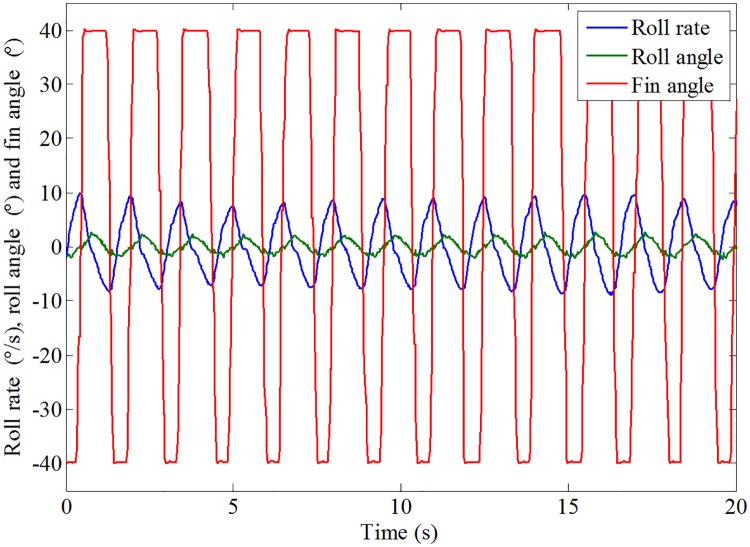
Roll response of the ship model and fin’s action with RRC.

The results of the roll response without and with roll reduction control (RRC) are shown in Figs 16 and 17, respectively. It can be seen that the roll motion of the ship model with RRC is greatly reduced. The root mean squares of the roll angle without and with RRC are 6.27° and 1.87°, respectively. The corresponding anti-rolling effect is about 70.18%, which is quite satisfactory. Therefore, the control form and strategy obtained in this paper is preliminarily verified. Fin stabilizers flapped in a trapezoidal form under the act of the proposed control strategy are promising and effective to reduce the roll motion at zero speed.

#### 4.3.2 Full scale roll reduction test

**Fig 18:**
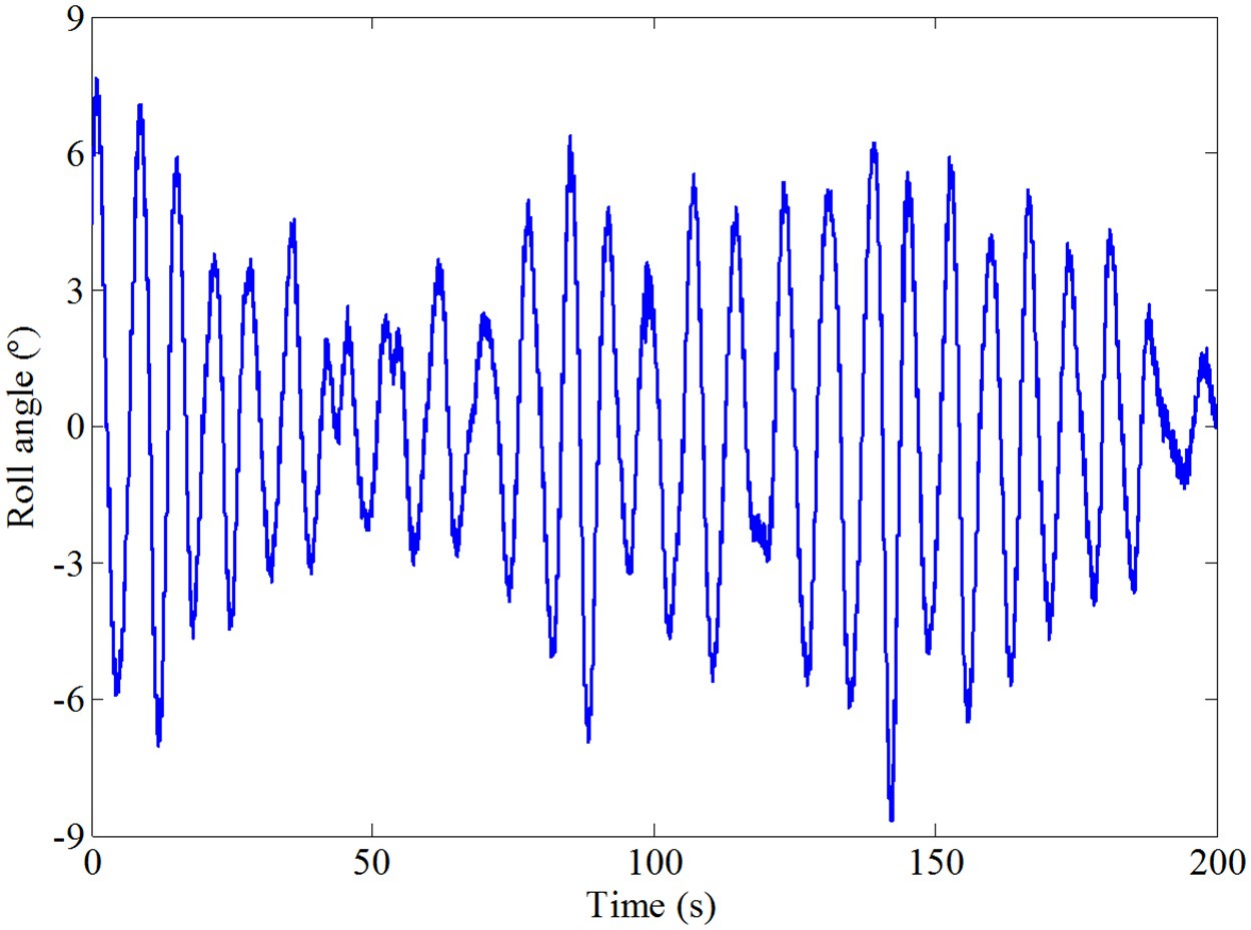
Typical roll motions experienced without RRC.

**Fig 19:**
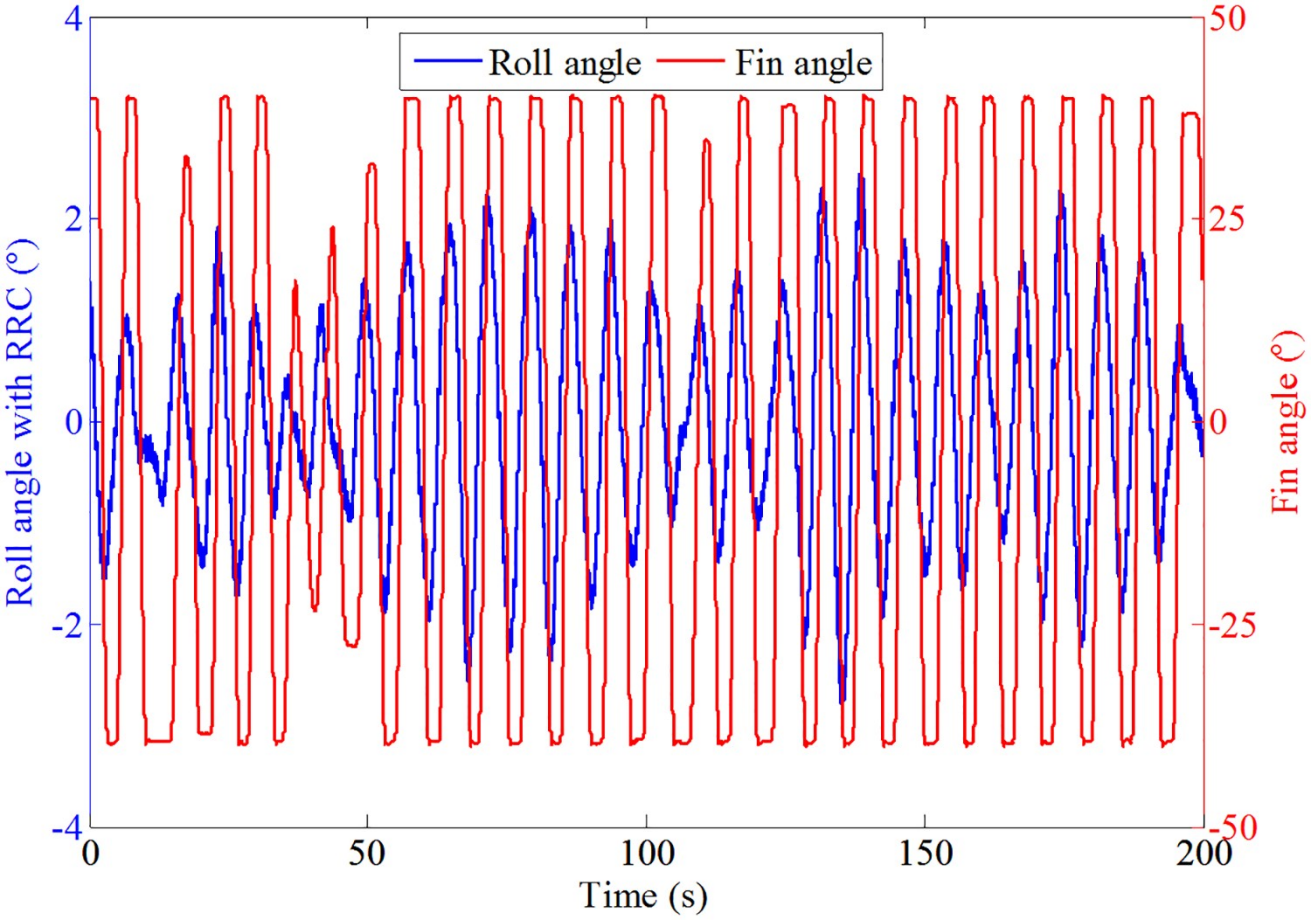
Roll response and fin’s action with RRC.

The full scale roll reduction test in at-anchor considitons was conducted after the completion of the fin stabilizer system modification with the obtained control form and method mentioned above based on the model tank test. During the full scale roll reduction test, the sea remained at around state 4. Typical roll motions which were experienced are shown in Fig 18. The RMS value of the roll angle without RRC is about 3.15°. Fig 19 shows the roll response and the fin’s action after the RRC is turned on. It can be seen that the roll amplitude with RRC has a greatly reduced. The corresponding RMS value of the roll angle can be calculated as 1.13°. The RMS value of the roll angle with RRC is reduced by 2.02° compared with that without RRC, and the anti-rolling effect is about 64.13%, which is quite satisfactory for the full scale roll reduction test. The results of the full scale test further verify the effectiveness and applicability of the obtained control form and strategy for fin stabilizers in at-anchor conditions.

## 4 Conclusion

In this paper, the control form of fin stabilizers at zero speed is investigated. Two kinds of fin angle control forms appearing in the existing literature, the sinusoidal and trapezoidal forms, are studied through a composite method of theoretical analysis and experimental research. The conclusions are drawn:

1. Based on the established reaction force model for fin stabilizer at zero speed, the reactions forces generated on the fin under the two control forms are compared and analyzed. The concept of half-cycle ratio is proposed for the trapezoidal form fin angle control. It is found that when the flapping amplitude is the same, the reaction force generated on the fin flapped in a trapezoidal form with a small half-cycle ratio is larger than that in sinusoidal form. Although the duration time of the reaction force generated on the fin flapped in sinusoidal form is longer than that in trapezoidal form, its overall effect is less than the trapezoidal one as it only produces a large force at its maximum slope. Therefore, the preliminary result is that the trapezoidal fin angle control form with a proper small half-cycle ratio is more suitable for fin stabilizers at zero speed.
2. The scaled model of an 84-meter-long fishery ship with two pairs of parallelogram fin stabilizers is selected as the research object to conduct the water tank test to verify the results obtained from theoretical analysis. The forced rolling tests with fin stabilizers flapped in sinusoidal and trapezoidal forms (with half-cycle ratio of 0 ∼ 0.5) of different amplitudes were conducted. It is found that the forced rolling ability of the trapezoidal flapping is generally greater than that of the sinusoidal flapping, which is consistent with the theoretical analysis. The forced rolling ability increases with the increase of the fin’s flapping amplitude and the decrease of the half-cycle ratio. Therefore, the fins should be flapped in trapezoidal form with large flapping amplitude and small half-cycle ratio to generate large reaction force to improve the roll damping ability in at-anchor conditions.
3. Two kinds of control strategies for fin stabilizers at zero speed are obtained based on the results of the tank test. However, it is found that the two control methods are essentially the same in consideration of the physical limitations of the fin stabilizer actuation system. Finally, the control strategy for changing the flapping amplitude of the fins according to the roll magnitude of the ship is obtained through further analysis. The Kalman filter is adopted to predict the next roll peak according to the roll rate at the zero-crossing point of the roll angle to determine the following flapping amplitude of the fins. According to the obtained control logic, the model roll reduction tank test was conducted. A 70.14% anti-rolling effect is achieved under the obtained control form and strategy, and the effectiveness of the proposed method is preliminarily verified. The full scale zero-speed roll reduction test was also carried out. The anti-rolling effect reaches 64.13% at sea state 4, which further verifies the applicability and practicability of the obtained control form and strategy for fin stabilizers in at-anchor conditions.
